# The Preliminary Study of 16α-[18F]fluoroestradiol PET/CT in Assisting the Individualized Treatment Decisions of Breast Cancer Patients

**DOI:** 10.1371/journal.pone.0116341

**Published:** 2015-01-24

**Authors:** Yifei Sun, Zhongyi Yang, Yongping Zhang, Jing Xue, Mingwei Wang, Wei Shi, Beiling Zhu, Silong Hu, Zhifeng Yao, Herong Pan, Yingjian Zhang

**Affiliations:** 1 Department of Nuclear Medicine, Fudan University Shanghai Cancer Center, Shanghai, China; 2 Center for Biomedical imaging, Fudan University, Shanghai, China; 3 Department of Oncology, Shanghai Medical College, Fudan University, Shanghai, China; 4 Shanghai Engineering Research Center for Molecular Imaging Probes, Shanghai, China; Wayne State University, UNITED STATES

## Abstract

**Objective:**

To evaluate the clinical value of 16α-[18F]fluoroestradiol (18F-FES) PET/CT in assisting the individualized treatment decisions of breast cancer patients.

**Methods:**

Thirty-three breast cancer patients, who underwent both 18F-FES and 18F-FDG PET/CT from July 2010 to March 2013 in our center, were enrolled in this preliminary study. All the patients used 18F-FES PET/CT as a diagnostic tool with a clinical dilemma. We used the maximum Standardized Uptake Value (SUVmax) to quantify ER expression and a cutoff value of 1.5 to dichotomize results into ER positive and negative lesions. All patients were clinically followed up at least 6 months.

**Results:**

In evaluating equivocal lesions on conventional work-up group (n = 4), three lung lesions and another iliac lesion were enrolled. As for three lung lesions, 18F-FES PET/CT showed one lesion with high uptake, which suggested it was an ER positive metastasis. The other two lesions were 18F-FES negative, which meant an ER negative metastasis or secondary primary tumor. Additionally, one iliac lesion was detected by MRI. 18F-FDG uptake was high at the suspected lesion, whereas 18F-FES uptake was absent; In predicting origin of metastasis group (n = 2), two breast cancer patients had secondary primary tumors were collected. They were 18F-FES negative, which showed low possibility of metastasis from breast cancer and they were all confirmed by biopsy. In detecting ER status in metastasis group (n = 27), 18F-FES PET/CT showed increased 18F-FES uptake in all metastatic lesions in 11 patients; absent in all lesions in 13 patients; and the remaining 3 patients had both 18F-FES positive and negative lesions. Totally, on the basis of the 18F-FES PET/CT results, we found changes in the treatment plans in 16 patients (48.5%, 16/33).

**Conclusions:**

18F-FES PET/CT could assess the entire tumor volume receptor status; therefore, it may be used to assist the individualized treatment decisions of breast cancer patients.

## Introduction

Breast cancer is the most common nondermatologic cancer and the second leading cause of cancer death in women [1]. Over 70% of breast cancers are ER positive, and ER-directed adjuvant therapy is regarded as a crucial factor in decreasing the death rate of breast cancer [2]. However, not all the patients benefit from the endocrine therapy, and most initial responders later become intractable. And the objective response rate to second-line endocrine therapy is less than 20% [3]. One factor hypothesized to underlie the acquired resistance to endocrine therapy is discordancy of ER expression. 18%–55% of the patients present with discordant ER expression between primary tumor and metastasis [4, 5]. Because the ER status may not be consistent within the same patient, a single biopsy may not be representative of the ER status of the tumor burden as a whole [6]. In addition, ER expression may also change over time in the same patient, caused either by genetic or epigenetic loss of the receptor, such as ER promoter B associated factor 1 [7].

Positron emission tomography (PET) with ER-targeting radiopharmaceuticals is a noninvasive method for assessing regional ER expression in vivo. Several studies have shown that the detection of ER positive lesions by ^18^F-FES PET is reliable and that ^18^F-FES uptake correlates well with immunohistochemical scoring for ER [8–10]. Due to the discordant ER expression between primary tumor and metastatic lesions, clarifying the present ER status may be crucial to assisting the physicians in making individualized treatment decisions. Therefore, our preliminary study was aimed to evaluate whether ^18^F-FES PET/CT could be useful in clinical practice, especially for the patients with clinical dilemmas.

## Materials and Methods

### Patients Selection

From July 2010 to March 2013, 33 patients who had a history of ER positive breast cancer underwent both ^18^F-FES and ^18^F-FDG PET/CT in our center. The patients were enrolled through the following protocols: using ^18^F-FES PET-CT as a diagnostic tool for the patients presenting with clinical dilemmas. Concomitant bisphosphonate therapy or trastuzumab was not an exclusionary criterion but concurrent cytotoxic therapy or radiotherapy was. Also, any patient with suspicious liver lesions was not enrolled due to the high physiological uptake of ^18^F-FES in the liver. Meanwhile, ^18^F-FDG PET/CT were conducted within 7 days of ^18^F-FES PET-CT to assist in locating lesions.

The results of ^18^F-FES PET/CT of 33 patients were given to their clinical oncologists, and the treatment decisions were made by the multidisciplinary of breast cancer in our hospital.

Our protocol strictly followed the clinical study rules established by our review board, and informed written consent was obtained from all these enrolled patients.

### PET/CT Imaging

^18^F-FES was prepared according to published methods [11] and was modified by us, as reported in our prior study [12]. ^18^F-FDG was produced automatically by cyclotron (Siemens CTI RDS Eclips ST, Knoxville, TN) by using the Explora FDG4 module in our center. Additionally, ^18^F-FES and ^18^F-FDG PET/CT studies were not performed on the same day.

ER antagonists were discontinued for a minimum of 5 weeks before ^18^F-FES study to prevent false-negative results. The use of aromatase inhibitors was allowed. Approximately 6 mCi (222 MBq) of ^18^F-FES was administered intravenously over 1~2 minutes. Scanning was initiated 1 hour after administration of the tracer. The images were obtained on a Siemens biograph 16 HR PET/CT scanner. The transaxial intrinsic spatial resolution was 4.1 mm (full width at half maximum) in the center of the field of view. The data acquisition procedure was as follows: CT was first performed, from the proximal thighs to the head, with 120 kV, 80 to approximately 250 mA, pitch 3.6, rotation time 0.5. Immediately after the CT, a PET that covered the identical transverse field of view was obtained. Acquisition time was 2~3 minutes per table position. PET image data sets were reconstructed iteratively by applying the CT data for attenuation correction; coregistered images were displayed on a workstation.

Before the ^18^F-FDG PET/CT, all the patients were requested to fast for at least 4 hours. At the time of the tracer injection (dosage, 0.2 mCi/kg), the patients presented with a blood glucose level of less than 10 mmol/L. Before and after injection, the patients were kept lying comfortably in a quiet, dimly lit room. The parameters of PET/CT were the same as ^18^F-FES PET/CTs.

### Image Interpretation

A multimodality computer platform (Syngo; Siemens) was used for image review and manipulation. Two experienced nuclear medicine physicians evaluated the images for each patient independently. The reviewers reached a consensus in cases of discrepancy. Only FDG-avid lesions, which could be detected definitely by PET-CT, were included in the analysis.

Quantification of tumor metabolic activity was obtained by using the standardized uptake value (SUV) normalized to body weight and the maximum SUV (SUVmax) for each lesion was calculated. In reference to other ^18^F-FES PET studies, we used the SUVmax to quantify ER expression and a cutoff value of 1.5 to dichotomize results into ER positive negative lesions [10, 13]. Lesions smaller than 1.5 cm were excluded because of partial-volume limitation and resolution restriction.

### Follow up of the patients with clinical dilemmas

33 patients were classified into 3 subgroups according to their purpose of ^18^F-FES PET/CT: (1) Evaluating equivocal lesions on conventional work-up (n = 4)(conventional work-up included CT, MRI, ultrasound, ^18^F-FDG PET/CT and so on). Patients in this subgroup used ^18^F-FES PET/CT to identify whether the lesions were malignant or benign, and whether the malignant lesions were primary tumor or metastases; (2) Predicting origin of metastasis (n = 2); (3) Detecting ER status in metastatic patients (n = 27). All the patients were followed up for at least 6 months.

## Results

### Patient Characteristics

The characteristics of the 33 enrolled breast cancer patients were summarized in Table 1. All the patients were women and had a history of ER positive breast cancer.

**Table 1:** Patient Characteristics (n = 33).

**Characteristic**		**No. of patients**	**%**
Age, years	Mean	56	
	Range	29~78	
Purpose of FES PET	Assisting in clinical dilemma	33	100
	Evaluating equivocal lesions	4	12.1
	Predicting origin of metastasis	2	6.1
	Detecting ER status in metastasis	27	81.8
Primary tumor histology	Ductal	27	81.8
	Lobular	2	6.1
	Others	4	12.1
Immunology	ER positive	33	100
	PR positive	25	71.4
	Her2/neu positive	12	36.4
Prior adjuvant treatment	Chemotherapy	28	84.8
	Radiotherapy	16	48.5

### The Quality Control of ^18^F-FES

The radiochemical purity of ^18^F-FES prepared by us was over 98%. Specific activity was available for 33 scans, with a mean of 160,852GBq/mmol (range, 105,326–270,341GBq/mmol) at the time of injection.

### Diagnostic effect of ^18^F-FES in patients presenting with a clinical dilemma

**1**. **Evaluating equivocal lesions on conventional work-up**. Four patients underwent ^18^F-FES PET/CT to evaluate ambiguous findings on standard work-up.

**Fig 1:**
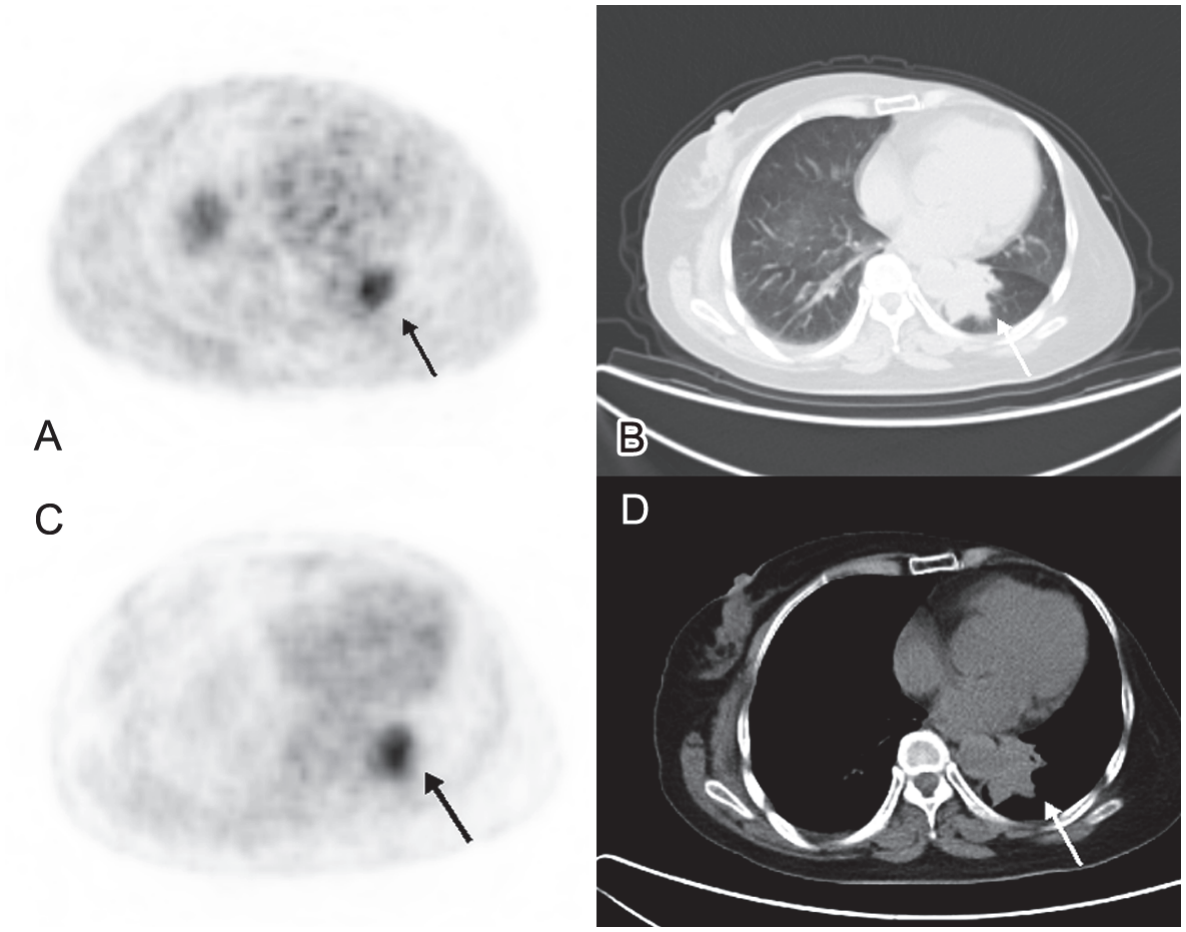
A 58-year-old female breast cancer patient. We detected a mass with irregular margin in left lung (B,D. CT imaging), with a maximum diameter of 4.5cm. It was difficult to differentiate it between metastasis and secondary primary lung cancer in CT imaging. The tumor presented with high uptake of both ^18^F-FES and ^18^F-FDG PET, SUVmax was 6.3 and 5.5 respectively. It suggested that it was a metastasis (A. ^18^F-FES PET/CT, C. ^18^F-FDG PET/CT). After operation, it was confirmed to be an ER positive metastasis from breast cancer.

Three lung lesions were found by CT, but it was difficult to differentiate them between metastasis and secondary primary lung cancers. Although ^18^F-FDG PET/CT showed high uptake in all three lesions, it could not distinguish between primary lung tumors and metastases from breast cancer either. ^18^F-FES PET/CT showed one lesion with high uptake, which suggested it was an ER positive metastasis (Fig. 1). The other two lesions were ^18^F-FES negative, which meant an ER negative metastasis or secondary primary tumor. After operation, the ^18^F-FES positive lesion was confirmed to be ER positive metastasis; one ^18^F-FES negative lesion was ER negative metastasis and the other one was a secondary primary lung cancer. Hence, histological evidence was concordant with all ^18^F-FES PET/CT results.

Additionally, one iliac lesion was detected by MRI and high uptake in ^18^F-FDG PET; however, neither MRI nor ^18^F-FDG PET was conclusive. ^18^F-FES uptake was absent at the suspected lesion, which meant it was not an ER positive lesion. Because her primary tumor was ER positive, and the patient had no obvious complaint; it was considered to be benign. Therefore, she did not receive the intended therapy, such as radiotherapy; and the lesion was closely observed. It indicated no metastatic evidence after 6 months follow up.

**Fig 2:**
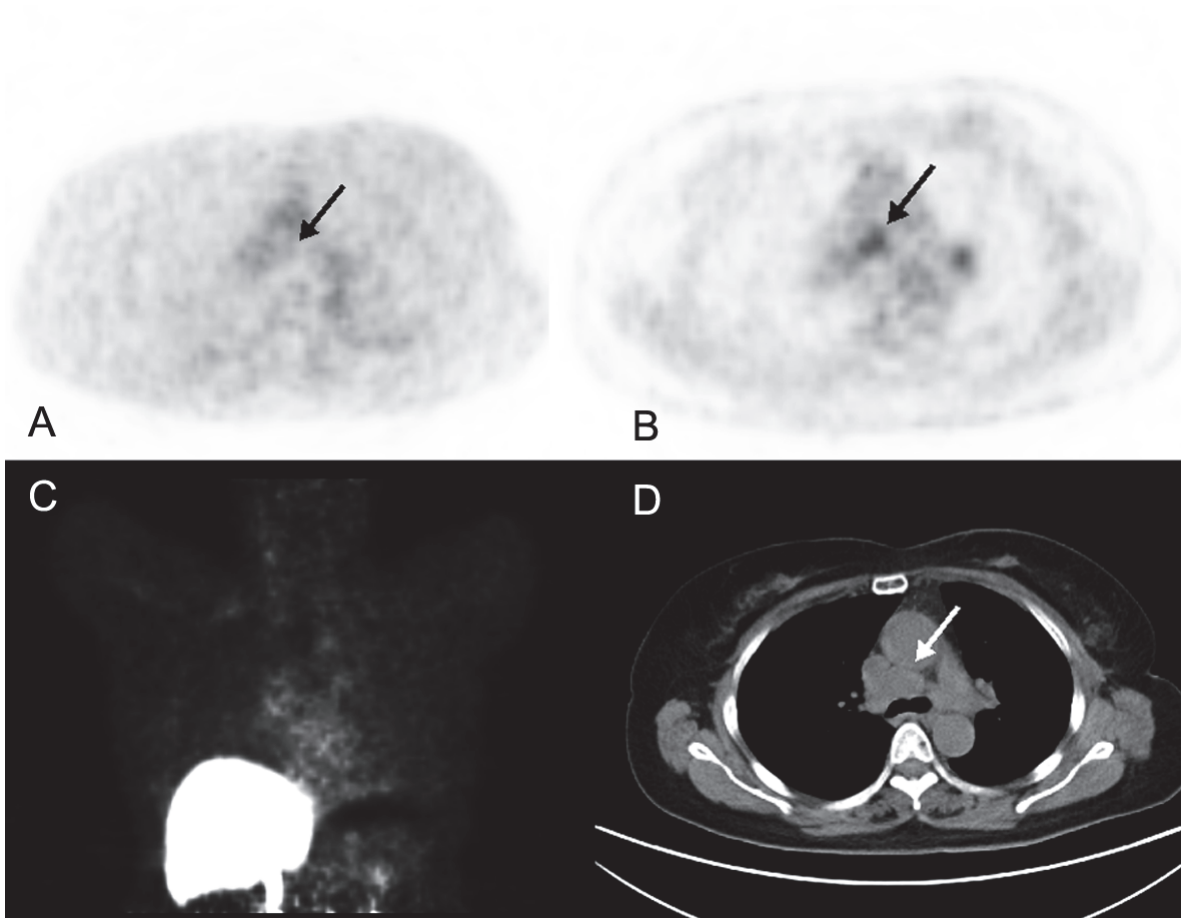
A 65-year-old female breast cancer patient, who also had renal clear cell carcinoma (RCCC), underwent ^18^F-FES and ^18^F-FDG PET/CT in our center. We detected a metastasis in mediastinal lymph node (D. CT imaging). The metastasis was no obvious ^18^F-FES uptake (A, C), whereas high ^18^F-FDG PET/CT uptake was detected (B. SUVmax = 3.9). The patient underwent an EBUS-TBNA after imaging. Histological evidence showed that the metastasis originated from RCCC.

**2**. **Predicting origin of metastasis**. Two breast cancer patients had secondary primary tumors (colon cancer and clear cell carcinoma of kidney, RCCC). Although ^18^F-FDG showed high uptake in all the metastases, it could not identify whether they came from breast cancer or the secondary primary tumor. One muscle lesion was found; however it was no ^18^F-FES uptake, which showed low possibility of metastasis from breast cancer; and it was confirmed by biopsy. In another one, we detected multiple enlarged mediastinal lymph nodes with no obvious ^18^F-FES uptake; and they were verified to be metastasis form RCCC after endobronchial ultrasound guided-transbronchial needle aspiration (Fig. 2).

**3**. **Detecting ER status in metastasis**. Twenty-seven patients underwent ^18^F-FES PET/CT to evaluate the ER status in recurrent or metastatic lesions. All the lesions showed high ^18^F-FDG uptake. The biopsies were not feasible in all of these patients because of the characteristics of the lesion (such as location) or the patients (such as comorbidity). Besides, all these patients had multiple lesions, which meant high possibility of heterogeneity; a single biopsy may not be representative.

In our study, ^18^F-FES PET/CT showed increased ^18^F-FES uptake in all metastatic lesions in 11 patients (category I); absent in all lesions in 13 patients (category II); and the remaining 3 patients had both ^18^F-FES positive and negative lesions (category III). According to our PET results, endocrine therapy was included in the comprehensive treatment of all patients in category I. In category II, the patients did not use endocrine therapy. In category III, two patients received endocrine therapy and one did not. ^18^F-FES PET/CT could assist the physicians in treatment-decision by detecting the present ER status.

**4**. **Clinical impact analysis**. On the basis of the ^18^F-FES PET/CT results, we found changes in the treatment plans in 16 patients (48.5%, 16/33). Thirteen patients averted unnecessary endocrine therapy due to detecting ^18^F-FES negative lesions. Two patients’ chemotherapy regimes may change by clarifying that they were not metastasis from breast cancer but from a secondary primary tumor after ^18^F-FES imaging. One patient avoided unnecessary radiotherapy because ^18^F-FES PET/CT considered it was a benign lesion.

## Discussion

ER expression is routinely measured in clinical practice by in vitro assay of biopsy material. Although it is the strongest predictor of response to hormonal treatment, it is far from perfect. The limitations could be concluded as follows: The technique is semi-quantitative, which can result in inter-observer variation, and ER scoring depends on the antibody used and delay-to-fixation time [14, 15]. The recent systematic review by the American Society of Clinical Oncology and the College of American Pathologists revealed that up to 20% of all immunohistochemical determinations worldwide are inaccurate [16]. Furthermore, the ER status in recurrent or metastatic lesions might not be consistent with the primary tumors after adjuvant therapy, as reported in our prior study [17]. According to these studies, ^18^F-FES PET detected discordant ER expression in 10%~37% breast cancer patients [2, 18, 19]. Furthermore, Sundararajan et al [20] had mentioned that an objective response was seen in only 7% to 21% of previously treated patients. As these reasons mentioned above, the results of the baseline ^18^F-FES PET analysis might provide current information about tumor expression of ER in the recurrent or metastatic lesions and, therefore, might predictive for clinical benefit from endocrine therapy more accurately than pathologic assessment of the primary tumors. Hence, we strongly suggested that the recurrent or metastatic breast cancer patients undergo ^18^F-FES PET/CT before receiving endocrine therapy, which might avoid some unnecessary endocrine strategy and its accompanying adverse reaction.

In most cases, ^18^F-FDG PET/CT could identify whether the lesions were malignant or benign. However, it could be inconclusive sometimes. Moreover, ^18^F-FDG PET/CT could not differentiate between metastases from different tumor types and detect ER status in metastases originating from breast cancer.

Four patients with ambiguous lesions underwent ^18^F-FES PET/CT to establish a diagnosis in the case of equivocal conventional work-up. We found that ^18^F-FES PET/CT could be used to prove the presence of ER positive metastases in the case of an equivocal conventional work-up. One ER positive metastasis was confirmed and one benign lesion was excluded by ^18^F-FES PET/CT; besides, one secondary primary tumor was considered due to low ^18^F-FES uptake. However, ^18^F-FES PET/CT cannot be used to exclude metastases in all cases because the heterogeneity exists; for instance, we detected one ER negative metastasis, and of course, no ^18^F-FES uptake was found.

With the development of modern cancer care, the incidence of secondary primary tumor increased during the past decades [21]. In our study, two patients had secondary primary cancers, they referred to undergo ^18^F-FES PET/CT in order to predict origin of metastasis; and the results showed that ^18^F-FES PET/CT may be useful. The metastatic lesions with low ^18^F-FES uptake were more likely from a secondary primary tumor than breast cancer.

In light of a possible conversion in ER phenotype, knowledge of ER expression can potentially affect making treatment-decision whether to use endocrine therapy.^18^F-FES PET has been shown to detect ER-positive metastasis with high specificity [8–10]. Peterson LM et al [22] found that 100%(2/2) patients with low FES standard uptake value tumors would have progressive disease within 6 months after hormonal therapy and 67%(2/3) patients with qualitatively FES-negative tumors would not get benefit from endocrine therapy. Therefore, this technique may be used as a surrogate for biopsy when lesions are difficult to access. In our study, 27 patients underwent ^18^F-FES PET/CT for detecting present ER status. In them, ^18^F-FES PET/CT showed increased ^18^F-FES uptake in all metastatic lesions in 11 patients; absent in all lesions in 13 patients; and the remaining 3 patients had both ^18^F-FES positive and negative lesions. Due to the absence of ^18^F-FES uptake in all of the lesions in 13 patients, they did not receive endocrine therapy. This meant clinical oncologists in our center had noticed that the heterogeneity of ER expression and switched their therapy management upon the results of ^18^F-FES PET/CT. ^18^F-FES PET has clear potential to improve therapeutic decision making [23].

However, the present study had several limitations. The first is the limited number of patients and it was only a preliminary study, it cannot demonstrate our result sufficiently. Another limitation was that all the patients had malignant tumors, it was impossible for us to obtain immunohistochemistry assay of biopsy material in most patients, we could not compare ^18^F-FES uptake with the golden criteria. Third, because of the heterogeneity of ER expression, no ^18^F-FES uptake cannot be used to exclude metastasis in all cases; it might still be inconclusive in some patients with clinical dilemmas. Therefore, we strongly recommended that a prospective, multi-center study should be performed to further investigate the clinical impact of ^18^F-FES PET/CT.

## Conclusion

Our preliminary study with limited number of patients showed that ^18^F-FES PET/CT, as a noninvasive modality, could assess the entire tumor volume receptor status; therefore, it may be used to assist the individualized treatment decisions of breast cancer patients.
